# Supplementary material for: A multi-criteria decision-making framework for managing the safety of marine recreational powered platforms: Integration with the SHELL model
Source: PLoS One. 2025 Aug 22;20(8):e0330820. doi: 10.1371/journal.pone.0330820 (PMC12373208; doi:10.1371/journal.pone.0330820)
Supplement: S1 File — (DOCX) [file pone.0330820.s001.docx]

|  | GA1 | GA2 | GA3 | CR1 | CR2 | CR3 | AD1 | AD2 | AD3 | MEAN | SD | CV |
| --- | --- | --- | --- | --- | --- | --- | --- | --- | --- | --- | --- | --- |
| S1 | 4 | 5 | 5 | 5 | 4 | 4 | 5 | 5 | 4 | 4.56 | 0.53 | 0.12 |
| S2 | 5 | 5 | 4 | 5 | 4 | 4 | 4 | 5 | 5 | 4.56 | 0.53 | 0.12 |
| S3 | 2 | 4 | 3 | 5 | 2 | 3 | 3 | 4 | 4 | 3.33 | 1.00 | 0.3 |
| S4 | 3 | 5 | 4 | 5 | 2 | 2 | 3 | 5 | 4 | 3.67 | 1.22 | 0.33 |
| S5 | 3 | 4 | 4 | 5 | 2 | 3 | 3 | 5 | 4 | 3.67 | 1.00 | 0.27 |
| H1 | 3 | 5 | 4 | 5 | 4 | 3 | 4 | 4 | 4 | 4.00 | 0.71 | 0.18 |
| H2 | 2 | 5 | 5 | 5 | 3 | 4 | 4 | 4 | 5 | 4.11 | 1.05 | 0.26 |
| H3 | 3 | 5 | 5 | 5 | 4 | 2 | 4 | 3 | 5 | 4.00 | 1.12 | 0.28 |
| H4 | 3 | 5 | 4 | 5 | 4 | 4 | 4 | 4 | 4 | 4.11 | 0.60 | 0.15 |
| H5 | 3 | 4 | 4 | 5 | 3 | 3 | 3 | 4 | 4 | 3.67 | 0.71 | 0.19 |
| E1 | 4 | 4 | 4 | 4 | 3 | 4 | 4 | 5 | 4 | 4.00 | 0.50 | 0.13 |
| E2 | 4 | 5 | 4 | 4 | 4 | 4 | 4 | 5 | 4 | 4.22 | 0.44 | 0.10 |
| E3 | 4 | 5 | 4 | 4 | 3 | 5 | 4 | 5 | 5 | 4.33 | 0.71 | 0.16 |
| E4 | 3 | 5 | 5 | 5 | 3 | 4 | 4 | 4 | 5 | 4.22 | 0.83 | 0.20 |
| E5 | 3 | 4 | 3 | 4 | 3 | 4 | 3 | 4 | 4 | 3.56 | 0.53 | 0.15 |
| L1 | 5 | 5 | 4 | 5 | 5 | 5 | 4 | 5 | 4 | 4.67 | 0.50 | 0.11 |
| L2 | 5 | 5 | 4 | 5 | 3 | 3 | 3 | 4 | 4 | 4.00 | 0.87 | 0.22 |
| L3 | 2 | 3 | 4 | 5 | 3 | 4 | 3 | 4 | 5 | 3.67 | 1.00 | 0.27 |
| L4 | 3 | 5 | 4 | 5 | 3 | 4 | 5 | 5 | 5 | 4.33 | 0.87 | 0.20 |
| L5 | 3 | 5 | 5 | 5 | 3 | 3 | 4 | 5 | 5 | 4.22 | 0.97 | 0.23 |
| TOTAL | | | | | | | | | | 80.89 |  | |
| AVG. | | | | | | | | | | 4.04 |  | |

S1 File. Survey data and result analysis for each criterion from 9 experts.

This file comprises three representatives each from government agencies (GA), the private sector (CR), and academia (AD). Emergency Response and Safety Knowledge (L1) received the highest average score, whereas Standard Guidelines for Operation (S3) received the lowest.
