# Supplementary material for: A multi-criteria decision-making framework for managing the safety of marine recreational powered platforms: Integration with the SHELL model
Source: PLoS One. 2025 Aug 22;20(8):e0330820. doi: 10.1371/journal.pone.0330820 (PMC12373208; doi:10.1371/journal.pone.0330820)
Supplement: S2 File — (DOCX) [file pone.0330820.s002.docx]

**DEMATEL Questionnaire**

1. Instruction：Instructions for Completing the Influence Relationship Matrix: The response scale ranges from “0” to “4”：

“0” indicates no influence.

“1” indicates very slight influence.

“2” indicates low influence.

“3” indicates moderate influence.

“4” indicates high influence.

1. Questionnaire：

|  | S1 | S2 | H2 | H4 | E2 | E3 | E4 | L1 | L4 | L5 |
| --- | --- | --- | --- | --- | --- | --- | --- | --- | --- | --- |
| S1 |  |  |  |  |  |  |  |  |  |  |
| S2 |  |  |  |  |  |  |  |  |  |  |
| H2 |  |  |  |  |  |  |  |  |  |  |
| H4 |  |  |  |  |  |  |  |  |  |  |
| E2 |  |  |  |  |  |  |  |  |  |  |
| E3 |  |  |  |  |  |  |  |  |  |  |
| E4 |  |  |  |  |  |  |  |  |  |  |
| L1 |  |  |  |  |  |  |  |  |  |  |
| L4 |  |  |  |  |  |  |  |  |  |  |
| L5 |  |  |  |  |  |  |  |  |  |  |

|  | S1 | S2 | H2 | H4 | E2 | E3 | E4 | L1 | L4 | L5 |
| --- | --- | --- | --- | --- | --- | --- | --- | --- | --- | --- |
| S1 |  |  |  |  |  |  |  |  |  |  |
| S2 |  |  |  |  |  |  |  |  |  |  |
| H2 |  |  |  |  |  |  |  |  |  |  |
| H4 |  |  |  |  |  |  |  |  |  |  |
| E2 |  |  |  |  |  |  |  |  |  |  |
| E3 |  |  |  |  |  |  |  |  |  |  |
| E4 |  |  |  |  |  |  |  |  |  |  |
| L1 |  |  |  |  |  |  |  |  |  |  |
| L4 |  |  |  |  |  |  |  |  |  |  |
| L5 |  |  |  |  |  |  |  |  |  |  |
