# Supplementary material for: A multi-criteria decision-making framework for managing the safety of marine recreational powered platforms: Integration with the SHELL model
Source: PLoS One. 2025 Aug 22;20(8):e0330820. doi: 10.1371/journal.pone.0330820 (PMC12373208; doi:10.1371/journal.pone.0330820)
Supplement: Attachment — Submitted filename: Response_to_Reviewers_auresp_2.docx [file pone.0330820.s004.docx]

**Additional Editor Comments Reply**

**Comment #1：**

• While improved, the abstract could be further enhanced by highlighting the unique contribution of the SHELL + DEMATEL integration in a single sentence near the end. Currently, it reads more like a summary than a pitch.

**Authors’ Reply: Thanks for the valuable comment.** **In response to this suggestion, we have added the following sentence to the abstract section of the manuscript to highlight the unique contribution and methodological innovation of this study："Building on the above findings, this study confirms the effectiveness of an innovative integration of the SHELL model and the DEMATEL method, which provides a structured and adaptive framework capable of systematically identifying systemic navigational risks in marine recreational activities."**

**(Please refer to the revised manuscript, pages ii, iii.)**

**Comment #2：**

• Although a section is dedicated to discussing applicability to Southeast Asia and the Pacific Islands, the analysis is brief. Consider adding a short comparative note (1–2 sentences) on how regulatory gaps in one or two specific countries mirror Taiwan’s situation, with references if possible.

**Authors’ Reply: Thanks for the valuable comment. In addition to discussing the applicability of the research framework to Southeast Asia and Pacific Island nations, we further highlighted the similarities and regulatory gaps between these countries and Taiwan (Please refer to the revised manuscript, pages 41, 42). To enhance the completeness of the discussion, six additional references were incorporated (Please refer to the revised manuscript, page 51).**

**Comment #3：**

• Tables 4 and 7 are informative, but they are densely formatted. Adding clearer headings or footnotes for “centrality” and “net effect” values could improve reader comprehension.

**Authors’ Reply: Thanks for the comment. We have revised the titles of Tables 4 and 7 to enhance clarity (Please refer to the revised manuscript, pages 24, 28) and added a footnote to Table 4 (Please refer to the revised manuscript, page 24) to improve reader comprehension.**
